# Domestication and Genetic Improvement Alter the Symbiotic Microbiome Structure and Function of Tomato Leaf and Fruit Pericarp

**DOI:** 10.3390/microorganisms12071351

**Published:** 2024-07-02

**Authors:** Fei Li, Hongjun Lyu, Henan Li, Kuanling Xi, Yin Yi, Yubin Zhang

**Affiliations:** 1 Key Laboratory of Biodiversity Conservation in Karst Mountain Area of Southwest of China, Forestry Ministry, School of Life Sciences, Guizhou Normal University, Guiyang 550001, China; hongjunlv2008@126.com (H.L.); xkl18386126037@outlook.com (K.X.); gzklppdr@gznu.edu.cn (Y.Y.); yubinzhang2@outlook.com (Y.Z.); 2 Shandong Province Key Laboratory for Biology of Greenhouse Vegetables, Institute of Vegetables, Shandong Academy of Agricultural Sciences, Shandong Branch of National Improvement Center for Vegetables, Huang-Huai-Hai Region Scientific Observation and Experimental Station of Vegetables, Ministry of Agriculture and Rural Affairs, Jinan 250100, China

**Keywords:** tomato, domestication, symbiotic microbiomes, bacillus, GWAS

## Abstract

Many studies have attempted to explore the changes in the structure and function of symbiotic microbiomes, as well as the underlying genetic mechanism during crop domestication. However, most of these studies have focused on crop root microbiomes, while those on leaf and fruit are rare. In this study, we generated a comprehensive dataset including the metagenomic (leaf) and metatranscriptomic (fruit pericarp in the orange stage) data of hundreds of germplasms from three tomato clades: *Solanum pimpinellifolium* (PIM), cherry tomato (*S. lycopersicum* var. cerasiforme) (CER), and *S. lycopersicum* group (BIG). We investigated the effect of domestication and improvement processes on the structure of the symbiotic microbiome of tomato leaf and fruit pericarp, as well as its genetic basis. We were able to obtain the composition of the symbiotic microbiome of tomato leaf and fruit pericarp, based on which the tomato clade (PIM, CER, or BIG) was predicted with high accuracy through machine learning methods. In the processes of tomato domestication and improvement, changes were observed in the relative abundance of specific bacterial taxa, Bacillus for example, in the tomato leaf and fruit pericarp symbiotic microbiomes, as well as in the function of these symbiotic microbiomes. In addition, SNP loci that were significantly associated with microbial species that are characteristic of tomato leaf were identified. Our results show that domestication and genetic improvement processes alter the symbiotic microbiome structure and function of tomato leaf and fruit pericarp. We propose that leaf and fruit microbiomes are more suitable for revealing changes in symbiotic microbiomes during the domestication process and the underlying genetic basis for these changes due to the exclusion of the influence of environmental factors such as soil types on the microbiome structure.

## 1. Introduction

Our understanding of microbes has undergone a fundamental paradigm shift over the past two decades. It is now recognized that eukaryotes are meta-organisms and must be viewed as inseparable functional units along with microorganisms [1]. The plant microbiome is one of the key determinants of plant health and productivity. It can promote multiple plant holobiont functions [2,3,4], such as (i) seed germination and growth, (ii) nutrient supply, (iii) resistance to biotic stress (pathogen defense), (iv) resistance to abiotic stress, and (v) production of biologically active metabolites. The profound influence of the microbiome on its host, as well as the intensive plant–microbiome interactions, suggests that plants and their associated microbiomes are co-evolving [1]. Domestication and breeding greatly affect the diversity, abundance, and composition of plant microbiomes [5]. Some domesticated plants were found to have unique microbial community composition compared to their wild relatives [6,7].

Tomato (*Solanum lycopersicum*) is the most widely cultivated vegetable crop worldwide and an important source of human dietary fiber and nutrients. The breeding history of tomato mainly includes two stages: domestication and genetic improvement [8]. The wild currant tomato (*S. pimpinellifolium*, PIM) was domesticated to generate the cherry tomato (*S. lycopersicum* var. cerasiforme, CER), which was later improved to develop the cultivated tomato with larger fruits (*S. lycopersicum*, BIG) [9]. The effect of domestication and improvement processes on the community structure and function of tomato symbiotic microbiomes, especially in tomato fruits and leaves, has not been systematically studied.

Most microbiome studies rely solely on rRNA gene sequencing, which can introduce biases into the estimation of microbial diversity and abundance [10]. As an alternative, metagenomic sequencing offers a broader view of the microbiome’s composition and function, along with insights into microbe–host interactions [11]. These techniques enable the simultaneous processing of numerous samples, providing high-throughput capabilities ideal for extensive studies. Metagenomic and metatranscriptomic approaches offer a less biased and more accurate representation of natural microbial communities. By analyzing the microbiomes of genetically distinct plants in similar environmental conditions, researchers can determine the impact of host genetics on microbial community structure and function. Insights gained from these studies may lead to the development of microbial-based biocontrol strategies or the selection of crop varieties that naturally foster disease-suppressive microbial communities.

The rhizosphere microbiome of tomato has been extensively studied, mainly focusing on the mining of functional bacteria that can improve tomato biotic and abiotic stress resistance [12,13,14,15,16,17,18]. Little research has been carried out on the microbiome of the above-ground part of tomato plants. In the above-ground part, the microbiota is less influenced by the soil; therefore, plant genotype plays a more important role in symbiotic microbial selection [19,20].

In a previous study [9], we generated a comprehensive dataset including the genome and fruit pericarp transcriptome (in the orange stage, ~75% ripe) of hundreds of germplasms from PIM, CER, and BIG grown under the same cultivation conditions. The aims of the current study were to (i) understand the changes in the composition and function of the leaf and fruit microbiomes during tomato domestication and improvement, and (ii) obtain potential loci that regulate the leaf and fruit symbiotic microbiomes.

## 2. Materials and Methods

### 2.1. Genomic and Transcriptomic Data of Tomato

We previously established a dataset containing the genomic and transcriptomic data of hundreds of tomato germplasms. This dataset includes accessions of wild species and red-fruited tomato (*S. pimpinellifolium*, *S. lycopersicum* var. cerasiforme, and *S. lycopersicum*), representing various geographical origins, consumption types, and improvement statuses [9]. We selected the leaf genomic and fruit pericarp transcriptomic data from tomato accessions grown in the same environment and sequenced on the same platform to characterize the structure and function of their leaf and fruit microbiomes. As a result, we obtained the genomic data of 60 PIM accessions, 109 BIG accessions, 79 CER accessions, and 13 Wild tomato accessions as well as the transcriptomic data of 25 PIM accessions, 109 BIG accessions, 79 CER accessions, and 4 Wide accessions. The details including SRA numbers of these accessions are listed in Table S1.

### 2.2. Kraken Pipeline for Microbial Detection

The reads that failed to be mapped to the tomato reference genome were aligned to all known bacterial, archaeal, and viral microbial genomes using the ultrafast Kraken algorithm. We retrieved a total of 71,782 microbial genomes using the RepoPhlan platform (https://bitbucket.org/nsegata/repophlan, accessed on February 2023). Among these genomes, there were 5503 viral genomes and 66,279 bacterial or archaeal genomes. We constructed a comprehensive database consisting of 5503 viral genomes and 54,471 bacterial and archaeal genomes which possessed a quality score of 0.8 or higher [21].

The Kraken algorithm decomposes each sequencing read into k-mers (31-mers was used by default) and precisely matches each k-mer to the constructed microbial genome database (59,974 genomes), providing a putative taxonomy assignment of the lowest common ancestor for that read. Matching and classification using the Kraken algorithm are several orders of magnitude faster than performing direct genome alignments. The results were most accurate at the genus level. To remove the rarest species that may cause noise in the subsequent analysis, we retained the genera present in at least 10% [11] of the samples (leaf or fruit pericarp) from PIM, CER, and BIG.

### 2.3. Quantification of 16S from Abundant Genera

To validate the results obtained from the analysis of the tomato leaf metagenome, we amplified and quantified the 10 most abundant genera found in the tomato accessions. To carry this out, we planted 12 seeds per accession in a glasshouse environment and harvested the leaves. We combined four leaves from four plants to create one biological replicate, and we used a total of three biological replicates. Before extracting the DNA, we cleaned the leaves using a solution of 70% ethanol, 2% bleach, and water. The DNA extraction was performed using the CTAB method. The extracted DNA was then divided into equal concentrations (20 ng/μL) for the quantitative real-time PCR. The StepOnePlus™ Real-Time PCR System and SYBR(R) Green I dye were utilized for the amplification and quantification, following the manufacturer’s protocol (Applied Biosystems, Waltham, MA, USA). In brief, we used a final reaction volume of 10 μL with a primer concentration of 200 nM. We conducted tests on three biological replicates and three technical replicates. The comparative CT method setup was used with default parameters, allowing for a maximum of 40 cycles. We selected previously published primers for the following genera: *Clostridium* sp., *Pasteurella* sp., *Halothece* sp., *Candidatus* sp., *Bacillus* sp., *Halomonas* sp., *Hamiltonella* sp., *Synechococcus* sp., *Xanthomonas* sp., *Methylobacterium* sp. (Table S2). To facilitate comparisons, all samples were normalized to the V3-V4 region of the 16S rDNA.

### 2.4. Analysis and Comparison of Microbiome Structure

To compare the microbial composition of all tomato accessions, the read counts were normalized, and the heteroscedasticity was removed by using voom. The Krona tool was used for visualization. Mothur v1.30.2 (https://mothur.org/, accessed on February 2023) was used to calculate the richness and diversity indices (i.e., Chao, Shannon, Simpson, and ACE) of the leaf and fruit microbiomes based on the relative abundance data at the species level. Bray–Curtis distance matrices were calculated with QIIME v1.9.1 (http://qiime.org/install/index.html, accessed on February 2023) based on normalized species data to detect global variations in the composition of microbial communities. 

Partial least squares-discriminant analysis (PLS-DA), permutational multivariate analysis of variance (PERMANOVA) and analysis of similarities (ANOSIM) using the adonis function from the Vegan package (v2.5-3) were also performed to compare microbiome composition between samples. LEfSE (http://huttenhower.sph.harvard.edu/galaxy/, accessed on February 2023) was employed to identify distinguishing taxa among microbial communities at multiple levels and to visualize the results using taxonomic bar charts and cladograms [22].

### 2.5. Tomato Germplasm Differentiation Using Machine Learning

Gradient Boosting Machine (GBM) models were trained, automatically tuned, and validated using the GBM (v2.1.9) and Caret package (v6.0-94) in R (http://www.r-project.org/, accessed on March 2023). Random stratified sampling was used to divide all tomato samples into a training set (70%) and a validation set (30%). During model training, the data were first centered and normalized so that each sample had a mean of 0- or 1-unit standard deviation (SD). Two-fold cross-validation was used to create multiple subsets of the training set and perform search optimization of GBM parameters, including the interaction depth (1, 2, or 3) and tree number (50, 100, or 150), to maximize the area under the receiver operating characteristic curve (AUROC) of the final model. The learning rate (shrinkage) was fixed at 0.1, and the minimum observation tree per node was fixed at 5. In the case of imbalance in each class, upsampling was used to facilitate generalization of the model. The performance of the final model, including the ROC curve, PR curve, and confusion matrix (with a 50% threshold of recognition probability for class 1 and class 2), was assessed by applying the final model to the validation set. The ROC and PR curves, as well as the AUROV and AUPR values, were calculated using the PRROC package (v1.3.1), and the confusion matrix was calculated using the Caret package. Variable importance scores for the resulting non-zero model features were estimated using the GBM and Caret packages. The percentage contribution of a particular feature to the model’s prediction was estimated by dividing the variable importance score for that feature by the sum of all variable importance scores for a given model.

### 2.6. Identification of Regulatory Sites

To determine the relationship between microbial community composition and tomato genetic factors, we performed a genome-wide association study (GWAS) using the differential genera identified among PIM, CER, and BIG tomato leaf by the LEfSE analysis as phenotypes. The population structure of tomato accessions was determined by PLINK v1.9, the kinship matrices were calculated using MVP v1.0, and a GWAS was performed based on SNPs. A total of 2.65 million biallelic SNPs were identified across 239 tomato samples [9]. SNPs with a lower genotype rate (>95%) and minor allele frequency (MAF < 0.05) were excluded, and tomato samples with an idiotype deletion rate >= 5% were removed, resulting in a total of 210 samples for the GWAS analysis.

## 3. Results

### 3.1. Microbiome Structure of Tomato Leaf and Fruit Pericarp

Reads that cannot be mapped to the tomato reference genome from the sequencing data of tomato leaf and fruit pericarp samples can be assigned to bacterial and archaeal genomes [11,23]. An average of 2.02 ± 0.80% reads from the leaf samples belonged to bacterial or archaeal sequences, while the number was 0.92 ± 0.32% in the fruit samples (Figure S1). However, no significant difference was observed in the assigned read numbers among the PIM, CER, BIG, and Wide samples.

The accumulation curve (Figure S2) shows that most of the reads that could not be mapped to the tomato reference genome were assigned to corresponding microbial taxa. The number of bacterial genera reached a plateau of 1400 after the first 50 tomato accessions of BIG, CER, and PIM leaf and fruit pericarp samples had been analyzed (Figure S2), while the number bacterial genera obtained from the Wide samples was below the plateau. This result indicated that the obtained reads and characteristic profiles can represent the microbial characteristics of the PIM, CER, and BIG leaf and fruit pericarp samples, making them suitable for subsequent analyses.

**Figure 1:**
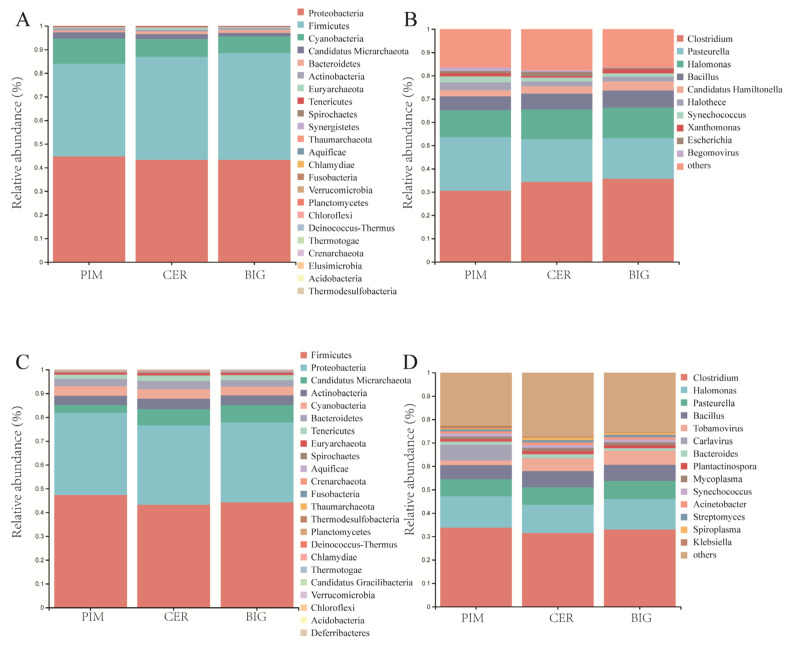
The microbial community composition of tomato leaf and fruit pericarp samples at the phylum and genus levels. (**A**) Microbiome community composition of tomato leaf at the phylum level; (**B**) microbiome community composition of tomato leaf at the genus level; (**C**) microbiome community composition of tomato fruit pericarp at the phylum level; (**D**) microbiome community composition of tomato fruit pericarp at the genus level.

The microbiome community composition of tomato leaf and fruit pericarp at the phylum and genus levels was investigated. Proteobacteria and Firmicutes were the most abundant bacterial phyla in both samples, together accounting for about 80% of the total relative abundance (Figure 1). In the tomato leaves, the most abundant phylum was Proteobacteria, followed by Firmicutes (Figure 1A). In the fruit pericarps, the most abundant phylum was Firmicutes, followed by Proteobacteria (Figure 1C). The bacterial phyla with higher abundance in the leaves and fruit pericarps also included Cyanobacteria, Candidatus, Micrarchaeota, Actinobacteria, Bacteroidetes, Euryarchaeota, and Tenericutes.

*Clostridium*, *Pasteurella*, *Halomonas*, *Bacillus*, *Halothece*, *Candidatus*, *Hamiltonella*, *Synechococcus*, and *Xanthomonas* were the most abundant bacterial genera in the leaf samples (Figure 1B), while *Clostridium*, *Alkaliphilus*, *Candidatus*, *Arthromitus*, *Geosporobacter*, *Mordavella*, *Oscillibacter*, and *Blautia* were the most abundant genera in the fruit pericarp samples (Figure 1D). The composition of leaf symbiotic microbiome was obtained using metagenome sequencing, while that of fruit pericarp was obtained using metatranscriptome sequencing. The differences in the microbial composition between the leaf and fruit pericarp samples might be ascribed to the different tissues (leaf and fruit); however, we could not exclude the possibility that different analysis methods used for these two types of data may also lead to such results.

Domestication and genetic improvement processes altered the relative abundance of symbiotic microbes in the tomato leaf samples at the phylum level. In the tomato leaves, the relative abundance of Firmicutes showed an increasing trend from PIM (38.50%) to CER (44.05%) to BIG (46.98%) (Figure 1A). Meanwhile, the relative abundance of Bacteroidetes increased, that of Cyanobacteria and Actinobacteria decreased, and that of Proteobacteria did not change significantly. Among the classes within Proteobacteria, the abundance of Gammaproteobacteria decreased during the domestication process, while that of Alphaproteobacteria and Betaproteobacteria increased (Figure 1A, Table S3).

The relative abundance of fruit pericarp symbiotic microorganisms also changed at the phylum level during tomato domestication and improvement. The relative abundance of Firmicutes in the BIG and CER fruits was lower than that of PIM fruits, while the relative abundance of Candidatus, Micrarchaeota, Actinobacteria, and Bacteroidetes was higher than that of PIM. Similar to the leaf symbiotic microbial composition, among the classes within Proteobacteria, the relative abundance of Gammaproteobacteria decreased during domestication, while that of Alphaproteobacteria and Betaproteobacteria increased (Figure 1C, Table S3).

We recognized that sample manipulation during DNA extraction or sequencing could potentially introduce microorganisms not typically found in tomato leaves. To exclude the possibility of artificially introducing abundant genera during sample collection, we employed quantitative PCR to detect 10 highly prevalent genera in tomato accessions (Table S2). We selected new plants under the assumption that the genera we identified are representative members of the tomato leaf microbiome. Remarkably, we successfully quantified the presence of all taxa and observed a consistent distribution across the accessions (Table S2). Notably, *Clostridium*, *Pasteurella*, and *Halomonas* remained the most abundant genera, effectively ruling out the likelihood of artificially introduced highly abundant genera.

### 3.2. Differences in Leaf and Fruit Pericarp Microbiome Structure among PIM, CER, and BIG

CER was domesticated from PIM and was later genetically improved to the cultivated BIG [9]. After domestication and genetic improvement, the yield, disease resistance, fruit size, and flavor of wild tomato all changed significantly. However, whether domestication and improvement processes also affect tomato leaf and fruit pericarp microbiome structure and function remains elusive.

**Figure 2:**
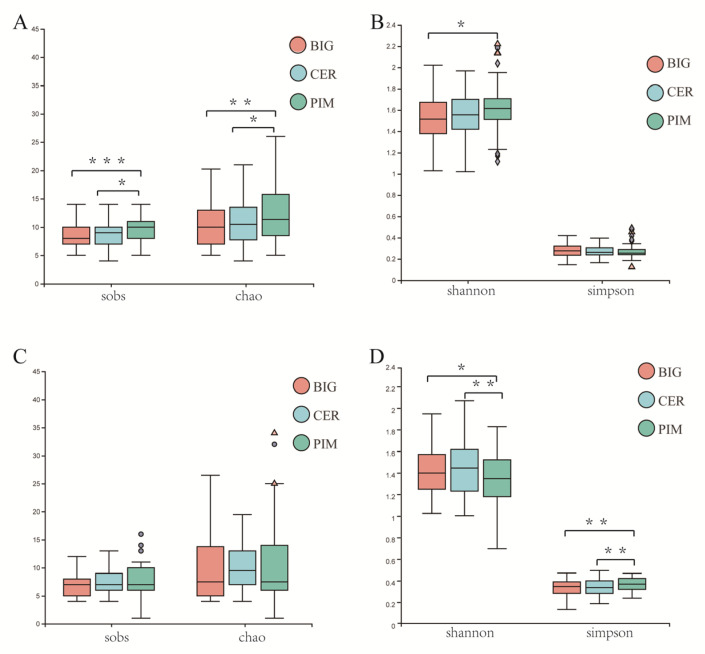
The effects of domestication and genetic improvement on the alpha diversity of the leaf and fruit pericarp microbiome. (**A**,**B**) Tomato leaf alpha diversity indices; (**C**,**D**) tomato fruit pericarp alpha diversity indices. Statistically significant differences were determined by ANOVA with Student’s *t*-test, *, *p*-value < 0.05; **, *p*-value < 0.01; ***, *p*-value < 0.001.

Domestication and genetic improvement altered the alpha diversity of the leaf microbiome. The diversity (Shannon index) and richness (sobs and Chao indices) of the PIM leaf microbiome were significantly higher than those of BIG (Figure 2A,B). The richness (sobs and Chao indices) of the PIM leaf microbiome was significantly higher than that of CER (Figure 2A), while the diversity (Shannon index) of the PIM leaf microbiome was also higher than that of CER but not statistically significant (Figure 2B). The richness and diversity of the CER leaf microbiome were higher than those of BIG, but the differences were not statistically significant (Figure 2A,B).

The effects of domestication and improvement on the alpha diversity of the fruit pericarp microbiome were not as significant as those on the leaf microbiome. The richness (sobs and Chao indices) of the PIM fruit pericarp microbiome was higher than that of BIG, but not statistically significant (Figure 2C). The diversity indices of (Shannon and Simpson) the PIM fruit pericarp microbiome were significantly higher (*p*-value = 0.009212 and 0.004476, respectively) than those of BIG (Figure 2D). Similarly, the diversity indices of the PIM fruit microbiome were significantly higher (*p*-value = 0.0411 and 0.008922, respectively) than those of CER (Figure 2D), while no significant difference was observed in richness (Figure 2C, sobs and Chao indices). The richness and diversity of the CER fruit microbiome were higher than those of BIG, but with no statistical significance (Figure 2C,D).

**Figure 3:**
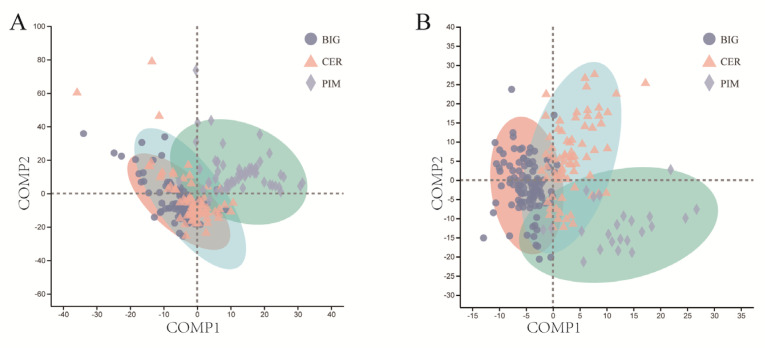
PIM, CER, and BIG tomato leaf and fruit pericarp microbiome community diversity and structure. Each dot represents a sample of tomato leaf or fruit pericarp, colored according to the tomato clade. (**A**,**B**) Partial least squares discriminant analysis (PLS-DA) for leaves and fruit pericarps, respectively.

To assess the similarity between microbial communities, principal coordinates analysis (PCoA) was performed based on the Bray–Curtis algorithm at the species level. The results showed no obvious separation among the three tomato clades for both the leaf and fruit pericarp microbial communities (Figure S3). Then, partial least squares discriminant analysis (PLS-DA), a supervised analysis suitable for high-dimensional data, was performed (Figure 3A,B). The leaf and fruit pericarp microbiomes of PIM, CER, and BIG were clustered separately, indicating significant differences in the overall structure of the microbial communities among the three tomato clades. The leaf microbiome of CER was located between those of PIM and BIG, and was closer to BIG; the same pattern was observed for the fruit pericarp microbiome of the three. This is consistent with the processes of tomato domestication (from PIM to CER) and genetic improvement (from CER to BIG). The *p*-values (*p*-value ≤ 0.001, Table S4) calculated from PERMANOVA and ANOSIM, based on the Bray–Curtis distance, further demonstrated the significant differences in the leaf and fruit microbiomes among the three tomato clades.

### 3.3. Random Forest Model Based on Microbial Community Composition Accurately Predicted Tomato Clade

**Figure 4:**
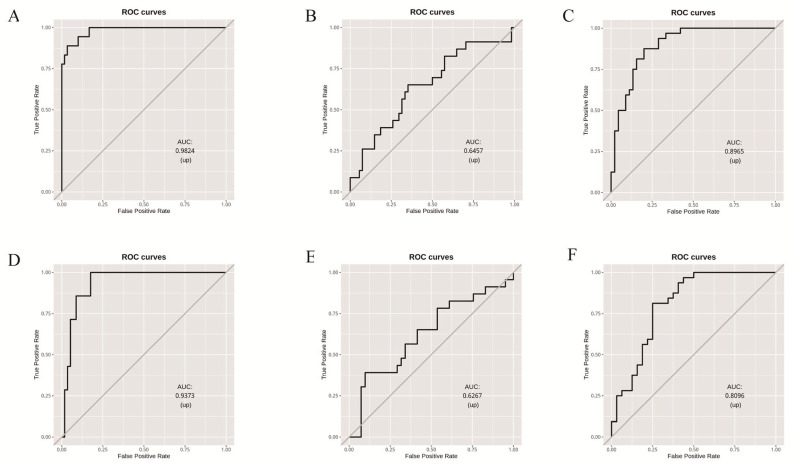
Random forest model based on microbial community composition accurately predicted tomato clade. (**A**–**C**), tomato leaf microbiome data analysis: (**A**) PIM 1-vs-all for tomato clade identification; (**B**) CER 1-vs-all for tomato clade identification; (**C**) BIG 1-vs-all for tomato clade identification. (**D**–**F**) tomato fruit pericarp microbiome data analysis, (**D**) PIM 1-vs-all for tomato clade identification; (**E**) CER 1-vs-all for tomato clade identification; (**F**) BIG 1-vs-all for tomato clade identification.

Stochastic gradient-boosting machine learning models were constructed to distinguish between tomato clades using the normalized data of leaf and fruit pericarp microbiomes, respectively, of PIM, CER, and BIG. The prediction performance of the model constructed based on both leaf and fruit pericarp microbiome data (model-both) was good, while the model constructed based on leaf microbiome data (model-leaf) outperformed the one using fruit pericarp microbiome data (model-fruit) (Figure 4 and Figure S4).

The sensitivity and specificity of the model were highest when predicting PIM, followed by BIG, with the poorest performance observed in predicting CER. The AUROC for model-leaf and model-fruit in predicting PIM were 0.9824 and 0.9373, respectively. For BIG, the corresponding values were 0.8965 and 0.8096, and for CER, they were 0.6457 and 0.6267. These results may be attributed to the transitional role of CER during the domestication and genetic improvement processes of tomatoes [8,9].

### 3.4. Differential Microbes in Leaf and Fruit Microbiomes of PIM, CER, and BIG

**Figure 5:**
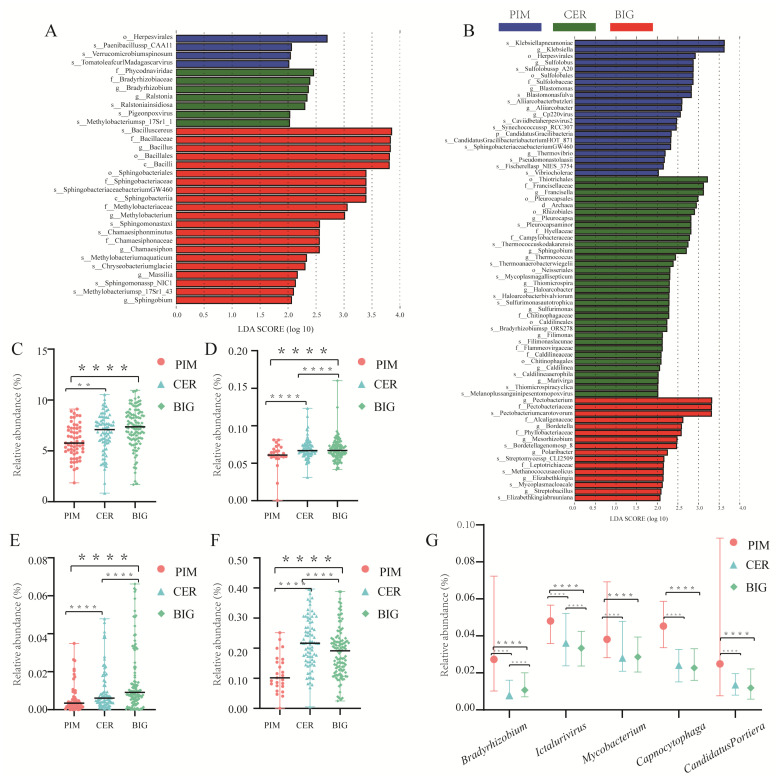
Microbial taxa whose abundance was altered by domestication and genetic improvement processes. (**A**) Tomato leaf microbiome. (**B**) Tomato fruit pericarp microbiome. (**C**) Tomato leaf *Bacillus* abundance increased from PIM to CER to BIG. (**D**) Tomato fruit pericarp *Bacillus* abundance increased from PIM to CER to BIG. (**E**) Tomato leaf *Sphingobium* abundance. (**F**) Tomato fruit pericarp *Sphingobium* abundance. (**G**) Tomato leaf *Bradyrhizobium*/*Ictalurivirus*/*Mycobacterium*/*Capnocytophaga*/*Candidatus Portiera* abundance alternation during domestication and genetic improvement. Statistically significant differences were determined by one-way ANOVA with Student’s *t*-test (*p*-value < 0.05). **, *p*-value < 0.01; ****, *p*-value < 0.0001.

The above observations indicated that the processes of tomato domestication (from PIM to CER) and genetic improvement (from CER to BIG) resulted in changes in the leaf and fruit microbiomes. PIM, CER, and BIG each possessed unique leaf and fruit microbiomes. Through an LEfSe analysis, we identified microbial taxa unique to PIM, CER, or BIG in both leaf metagenomic data and fruit pericarp metatranscriptomic data (Figure 5, Table S5).

The LEfSe analysis identified bacterial taxa specific to PIM, CER, and BIG in the tomato leaf and fruit pericarp microbiomes (FDR-adjusted *p*-value < 0.05, Wilcoxon rank-sum test; the absolute LDA > 2; Figure 5A,B, Table S5), the abundance of which was altered by domestication and improvement processes. Consistent with the transitional position of CER during tomato domestication and improvement, the relative abundance of many differential bacterial taxa of CER leaf and fruit pericarp microbiomes was between that of PIM and BIG (Figure 5). For example, the relative abundance of *Bacillus* in the leaf microbiome increased from 5.77% for PIM to 7.09% for CER, and then to 7.37% for BIG (*p*-value < 0.0001). The abundance of *Bacillus* in the fruit pericarps also increased during the processes of domestication and improvement, though it was not identified as a differential genus in the fruit pericarp microbiome by the LEfSe analysis (LDA = 4.84, *p*-value > 0.05) (Figure 5, Table S5).

In the leaf microbiome, the relative abundance of *Methylobacterium*, *Vibrio*, and *Chamaesiphon* increased, while that of *Plantactinospora*, *Paraburkholderia*, *Ictalurivirus*, *Capnocytophaga*, and *Candidatus Portiera*, decreased during the tomato domestication and improvement processes (Figure 5, Table S5). Although the above genera were present in the fruit microbiome and their abundances were also affected by tomato domestication and improvement processes, they were not considered as differential genera by LEfSe analysis (Supplementary Figure S5, Table S4). It is worth noting that the abundance of the differential genus *Sphingobium* identified in both leaf and fruit microbiomes showed an increasing trend from PIM to CER to BIG (Table S5).

### 3.5. Functional Profiling of the Tomato Leaf and Fruit Microbiomes

**Figure 6:**
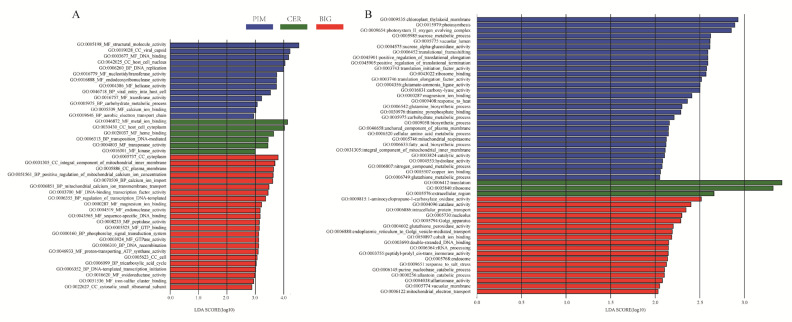
Bacterial functional categories whose abundance was altered by domestication and genetic improvement processes. (**A**) Tomato leaf microbiome. (**B**) Tomato fruit pericarp microbiome.

The processes of tomato domestication and improvement alter the leaf and fruit pericarp microbiome assembly. To elucidate whether changes in the microbiome assembly also alter the function of leaf and fruit microbiomes, we annotated the functional categories of the microbiomes (Table S6) and identified differential functional categories among PIM, CER, and BIG (Figure 6).

Transcription, translation, primary metabolism, flagellar assembly, and secondary metabolism (terpenoids, antibiotics, and xenobiotics) were the most enriched GO terms. The LEfSe analysis identified characteristic GO terms of the PIM leaf microbiome, including carbohydrate metabolic process (Figure 6, Table S6), and characteristic GO terms of the BIG leaf microbiome, including calcium ion import, magnet ion binding, iron–sulfur cluster binding, and proton-transporting ATP synthase activity.

The carbohydrate metabolic process was a characteristic GO term of the PIM fruit pericarp microbiome. In addition, the sucrose metabolic process, nitrogen compound metabolic process, cellular amino acid metabolic process, and glutamine biosynthetic process were characteristic GO terms of the PIM fruit microbiome. After domestication and improvement, the characteristic GO terms of BIG included cobalt ion binding, response to salt stress, and catalase activity.

### 3.6. GWAS Analysis Identifies Loci Regulating Tomato Symbiotic Microbiomes

**Figure 7:**
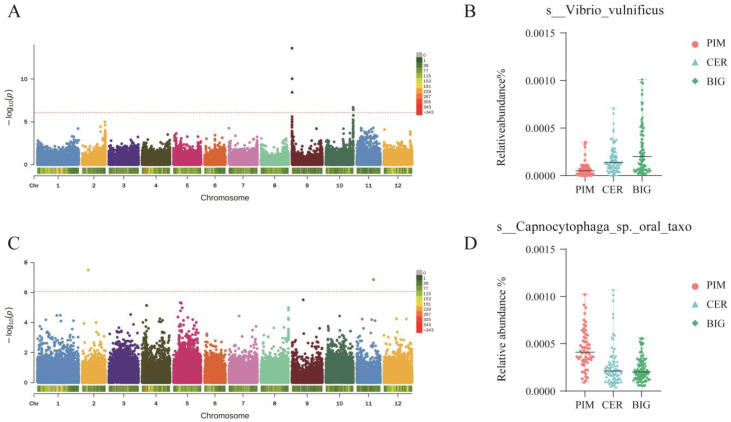
Tomato SNPs are associated with the microbiome structure. (**A**,**C**) Genome-wide association for specific microbial species. Left panel, Manhattan plot, using the tomato leaf dataset, indicated the major peaks (significant SNPs) distributed across twelve chromosomes, associated with microbial abundance of the specific microbial species. *p*-values were adjusted with FDR and values lower than 1 × 10^−6^ were consider significant (blue line). (**B**) Tomato leaf *Vibrio vulnificus* abundance increased from PIM to CER to BIG. (**D**) Tomato leaf *Capnocytophaga*_sp._oral_taxon_878 abundance decreased from PIM to CER to BIG.

To identify key loci that regulate the structure and composition of the tomato leaf microbiome, we performed a GWAS on the characteristic genera of the leaf microbiome of PIM, CER, and BIG, but no significantly associated SNPs were identified. Next, we performed a GWAS on the 40 specific microbial species identified by the LEfSe analysis (FDR-adjusted *p*-value < 0.05, Wilcoxon rank-sum test; absolute LDA score > 2; Table S7) in the three tomato clades. Twelve SNPs distributed on seven chromosomes (Figure 7, Table S8) were identified to be significantly associated (*p*-value < 1 × 10^−6^) with sixteen characteristic species in the leaf microbiome. Among these SNPs, one (SNP_9_1194) was located in the coding region of *Solyc09g005770*, but did not cause a frameshift mutation.

*Vibrio vulnificus* was the most differential bacterial species identified by LEfSe among PIM, CER, and BIG (LDA = 2.067426846, *p*-value = 9.52 × 10^−11^). *V. vulnificus*, widely known as a human pathogen, exists in marine ecosystems and serves as an endophyte and is symbiotic with seagrass such as *Zostera marina* [24]. Kwak et al. revealed the presence of *Vibrionaceae* in the tomato rhizosphere [12], while Park et al. reported that *V. vulnificus* could infect *Arabidopsis* and cause diseased phenotypes [25]. In this study, the relative abundance of *V. vulnificus* in the tomato leaf microbiomes increased during domestication, from 0.005% in PIM to 0.014% in CER, and finally to 0.02% in BIG. This change may be related to the reduced disease resistance during tomato improvement, but the role of *V. vulnificus* on tomato growth and development remains to be further studied.

The relative abundance of s__*Capnocytophaga*_sp._oral_taxon_878 was also altered during tomato domestication and improvement (LDA = 2.055804901, *p*-value = 3.29 × 10^−10^). Its relative abundance in the tomato leaf microbiome decreased during domestication, from 0.041% in PIM to 0.021% and 0.02% in CER and BIG, respectively. s__*Capnocytophaga*_sp._oral_taxon_878 belongs to the Flavobacteriaceae family, which has been widely reported to promote tomato growth and improve disease resistance [12,20,23].

Interestingly, 10 of the 12 significant SNPs appeared in more than one microbial as sociation analysis, indicating that specific regions on the tomato genome can affect the abundance of multiple microbial taxa. Among them, the SNP on chromosome 2 (SNP_2_56640) was significantly associated with the abundance of four microbial taxa in tomato. This SNP was located at chromosome 2:14,426,758 bp, and there was only one gene (*Solyc02g014020*) in the upstream and downstream 500-kb regions of this SNP. *Solyc02g014020* was annotated to the HAT family and encodes a transposon protein. However, its function is unknown.

## 4. Discussion

In this study, we analyzed the effects of domestication and improvement processes on the symbiotic microbiome structure of tomato leaves and fruit pericarps, as well as their genetic basis. The leaf microbiome was analyzed using metagenome sequencing to capture a broad snapshot of the genetic potential, while the pericarp microbiome was analyzed using metatranscriptome sequencing to focus on actively expressed genes, reflecting the dynamic response of the microbiome under fruiting conditions. Our results showed that those metagenomic and metatranscriptomic data that could not be aligned to the tomato reference genome contained sequences of the tomato leaf and fruit pericarp symbiotic microbiomes. Based on these sequences, we obtained the composition of symbiotic microbiomes of tomato leaf and fruit pericarp, and established models to accurately predict different tomato clades (PIM, CER, and BIG). The processes of tomato domestication and improvement have changed the structure of these symbiotic microbiomes. In addition, we also conducted GWAS analyses to identify SNPs significantly associated with characteristic microbial species of tomato leaf. Future studies are required to verify the underlying mechanisms of modulating the microbiome community for crop improvement purposes.

To reveal the changes in the structure and function of crop symbiotic microbial com- munities during crop domestication is of great practical significance. Many studies have been conducted to try to elucidate such changes; however, most of these studies focused on changes in the composition of root symbiotic microbial communities, while relatively little research has been carried out regarding leaf and fruit symbiotic microbial communities [1,26]. The processes of domestication and improvement have gradually reduced the richness and diversity of symbiotic microbial communities in tomato leaves and fruit pericarps (Figure 2). The effects of domestication on the alpha diversity of the rhizobacterial communities of various crops were inconsistent. The domestication process increased the rhizobacterial community diversity of maize and rice [27,28]. The alpha diversity of soybean and sunflower rhizobacterial communities decreased during domestication [29,30].

The structure of symbiotic microbes is affected by various factors such as the culture environment, host genotype, and plant compartment [27,31]. A comparison of the soil, rhizosphere, and seed microbiomes showed that soil bacterial community composition had a high impact on the bacterial community of the below-ground compartments (e.g., rhizosphere and root endosphere). However, the effect was progressively lowering from the rhizosphere to the root endosphere and finally to the seeds [19,20,32]. Therefore, the effects of domestication may not be equal for microbiota colonizing different plant compartments. Investigating the changes in the leaf or fruit pericarp microbiome structure during the domestication process may exclude the influence of environmental factors such as soil type on the microbiome structure, and may effectively reveal the changes in the plant symbiotic microbiome caused by domestication.

Members of *Bacillus* are known to have multiple beneficial traits which help the plants directly or indirectly through acquisition of nutrients, regulation of phytohormones, protection from pathogens and other abiotic stressors [33]. Bacterial seed endophytes of domesticated *Cucurbits* antagonizing leaf fungal and oomycete pathogens, as well as the majority of pathogen-suppressing endophytes belong to *Bacillus* [34]. During domestication and genetic improvement processes, the abundance of symbiotic *Bacillus* in the tomato leaves gradually increased (Figure 5C), from 5.77198% in PIM to 7.08867% in CER, and then to 7.36705% in BIG. The LEfSe analysis identified *Bacillus* as a biomarker genus in the BIG leaves. The abundance of the symbiotic *Bacillus* in tomato fruits also increased (Figure 5D), being 6.05%, 6.80%, and 6.85% in PIM, CER, and BIG fruits, respectively. *Bacillus* was not identified as a characteristic genus by the LEfSe analysis based on fruit transcriptomic data, probably due to the low amount of PIM transcriptomic data. The increase in the abundance of *Bacillus* over the domestication and improvement processes was also observed in the maize rhizobacterial communities [27]. Our data, as well as related studies with similar conclusions, indicate that the relative abundance of *Bacillus* increases during the processes of domestication and genetic improvement, which may be a common phenomenon in crops.

*Sphingobium* was identified as a differential taxon in both the tomato leaves and fruit pericarps, and its abundance showed an increasing trend from PIM to CER to BIG (Figure 5D,E). Studies based on *Arabidopsis* leaves showed that *Sphingobium* has the greatest potential to affect phyllosphere microbial community structure as keystone species [35]. *Sphingobium* is also one of the hubs in the microbial network of rice leaves [11]. The domestication and genetic improvement processes also changed the relative abundance of *Sphingobium* in the maize rhizosphere microbiome [27]. Domestication and breeding may select microbiomes that confer enhanced resistance to pathogens or abiotic stresses such as drought, salinity, and temperature extremes. This could be due to an increase in antagonistic microbes that inhibit pathogen growth through competitive exclusion or the production of antimicrobials. Microbes can improve plant water use efficiency or help in the synthesis of stress-protective compounds, thus aiding the plant under stress conditions.

Exploring the genetic mechanism of this phenomenon is of theoretical and practical significance. Based on leaf metagenomic data, we identified 16 SNPs that were significantly associated with differential microbial taxa during domestication. Most of these SNPs were located in the intergenic or noncoding regions (Table S8). Studies on the genes regulating the plant symbiotic microbiome are mainly focused on the root microbiome [36]. Veronica et al. [11] used leaf metagenomic data from more than 3000 rice germplasms to identify 22 SNPs associated with differential strains, of which 10 SNPs were located in the intergenic or noncoding regions. Studies on symbiotic microorganisms in tomato and rice leaves have shown that SNPs located in the intergenic or noncoding regions may play a key role in the regulation of symbiotic microbial structure. SNP on chromosome 2 (SNP_2_56640, located in the intergenic region) was significantly associated with the abundance of four microbial taxa in tomato (Figure 7). There was only one gene (*Solyc02g014020*) in the upstream and downstream 500 kb regions of this SNP. We speculated that this SNP may be involved in regulating the symbiotic microbiome of tomato leaf in a number of ways, including a. regulating *Solyc02g014020* gene expression and b. participating in the regulation of symbiotic microorganisms as non-coding RNAs [37]). In conclusion, the hypotheses and mechanisms discussed provide a framework for understanding how domestication and genetic improvement of tomato plants might influence their symbiotic microbiomes. These changes in the microbiome can have profound effects on plant health, stress tolerance, and productivity. Further research in this area can help optimize breeding strategies to not only improve plant traits but also to harness the benefits of beneficial plant–microbe interactions.

## 5. Conclusions

Plant breeding or genetic improvement could be used to intentionally modulate the composition of the microbiome and its function, recruiting disease antagonists and plant-growth promoters that enhance plant growth and health. The interactions of endophytic groups with the host plant and other microbial consortia on the physiology of the plant, however, are still poorly understood. Our study showed that the domestication and genetic improvement processes altered the structure and function of tomato leaf and fruit pericarp symbiotic microbiomes. We performed GWAS analyses to identify SNPs significantly associated with tomato leaf characteristic species. This lays the foundation for further exploration of the genetic mechanisms underlying the structural and functional changes of the symbiotic microbiome during crop domestication and genetic improvement.

## Data Availability

The data presented in this study are available in https://doi.org/10.1016/j.cell.2017.12.019.

## Supplementary Materials

The following supporting information can be downloaded at: https://www.mdpi.com/article/10.3390/microorganisms12071351/s1. Supplementary Figure S1. Percentage of reads assigned to bacterial and archaeal genomes in tomato leaf (**a**) and fruit (**b**) samples. Supplementary Figure S2. Accumulation curves of microbial species obtained from sequencing tomato leaf (**a**–**d**) and fruit (**e**–**h**) samples. Supplementary Figure S3. PIM, CER, and BIG tomato leaf and fruit pericarp microbiome community diversity and structure. Each dot represents a sample of tomato leaf or fruit pericarp, colored according to the tomato clade. Principal Coordinates Analysis (PCoA), leaf (**a**) and fruit pericarp (**b**) respectively. Supplementary Figure S4. Confusion matrix showing random forest models based on tomato leaf or fruit pericarp microbial community composition accurately predicted tomato clade. Supplementary Figure S5. Tomato fruit pericarp *Bradyrhizobium*/*Ictalurivirus*/*Mycobacterium*/*Capnocytophaga*/*CandidatusPortiera* abundance alternation during domestication and genetic improvement. Supplementary Table S1. The leaf metagenome and fruit pericarp metatranscriptome SRA numbers of tomato accessions (wild species and red-fruited tomato (*S. pimpinellifolium*, PIM, *S. lycopersicum* var. cerasiforme, CER, and *S. lycopersicum*, BIG). Supplementary Table S2. Quantitative PCR validation of metagenomic analysis. The table indicates the genera used for the experiments, followed by the primer sequences, the qPCR results (delta Ct and standard deviation). Then, the list of the 17 accessions and their groups are indicated. Supplementary Table S3. The microbial community composition of tomato leaf and fruit pericarp samples at the phylum and genus levels. Supplementary Table S4. PERMANOVA (Permutational multivariate analysis of variance) and ANOSIM (Analysis of similarities) analysis of tomato leaf and fruit pericarp microbial community composition. Supplementary Table S5. Specific microbial taxa to PIM, CER, and BIG in tomato leaf and fruit pericarp microbiomes identified through LEfSe (Linear discriminant analysis Effect Size) analysis (FDR-adjusted *p*-value < 0.05, Wilcoxon rank-sum test; the absolute LDA > 2). Supplementary Table S6. Specific GO functional categories to PIM, CER, and BIG in tomato leaf and fruit pericarp microbiomes identified through LEfSe (Linear discriminant analysis Effect Size) analysis (FDR-adjusted *p*-value < 0.05, Wilcoxon rank-sum test; the absolute LDA > 2). Supplementary Table S7. Specific microbial species to PIM, CER, and BIG in tomato leaf and fruit pericarp microbiomes identified through LEfSe (Linear discriminant analysis Effect Size) analysis (FDR-adjusted *p*-value < 0.05, Wilcoxon rank-sum test; the absolute LDA > 2). Supplementary Table S8. SNPs significantly associated with specific microbial species identified by LEfSe analysis.
