# Supplementary figures and images for: Domestication and Genetic Improvement Alter the Symbiotic Microbiome Structure and Function of Tomato Leaf and Fruit Pericarp
Source: Microorganisms. 2024 Jul 2;12(7):1351. doi: 10.3390/microorganisms12071351 (PMC11279011; doi:10.3390/microorganisms12071351)

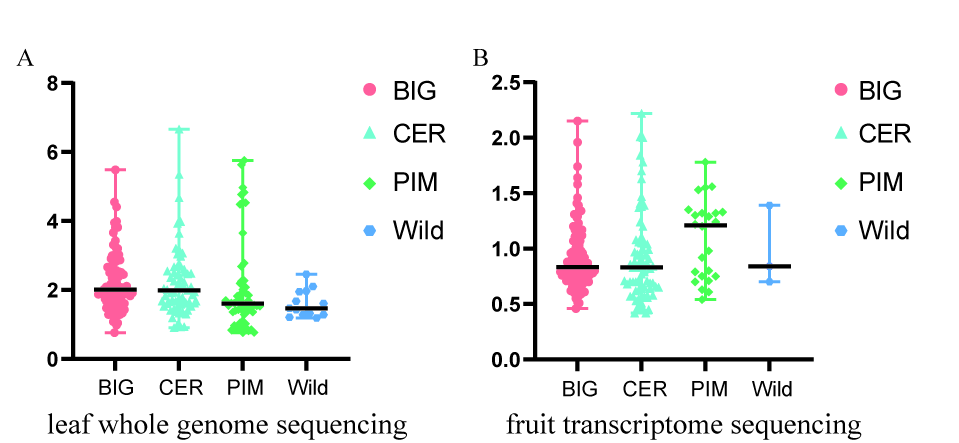

Supplement: Supplementary file 1 [file microorganisms-12-01351-s001.zip › Supplementary Figure S1.tif]

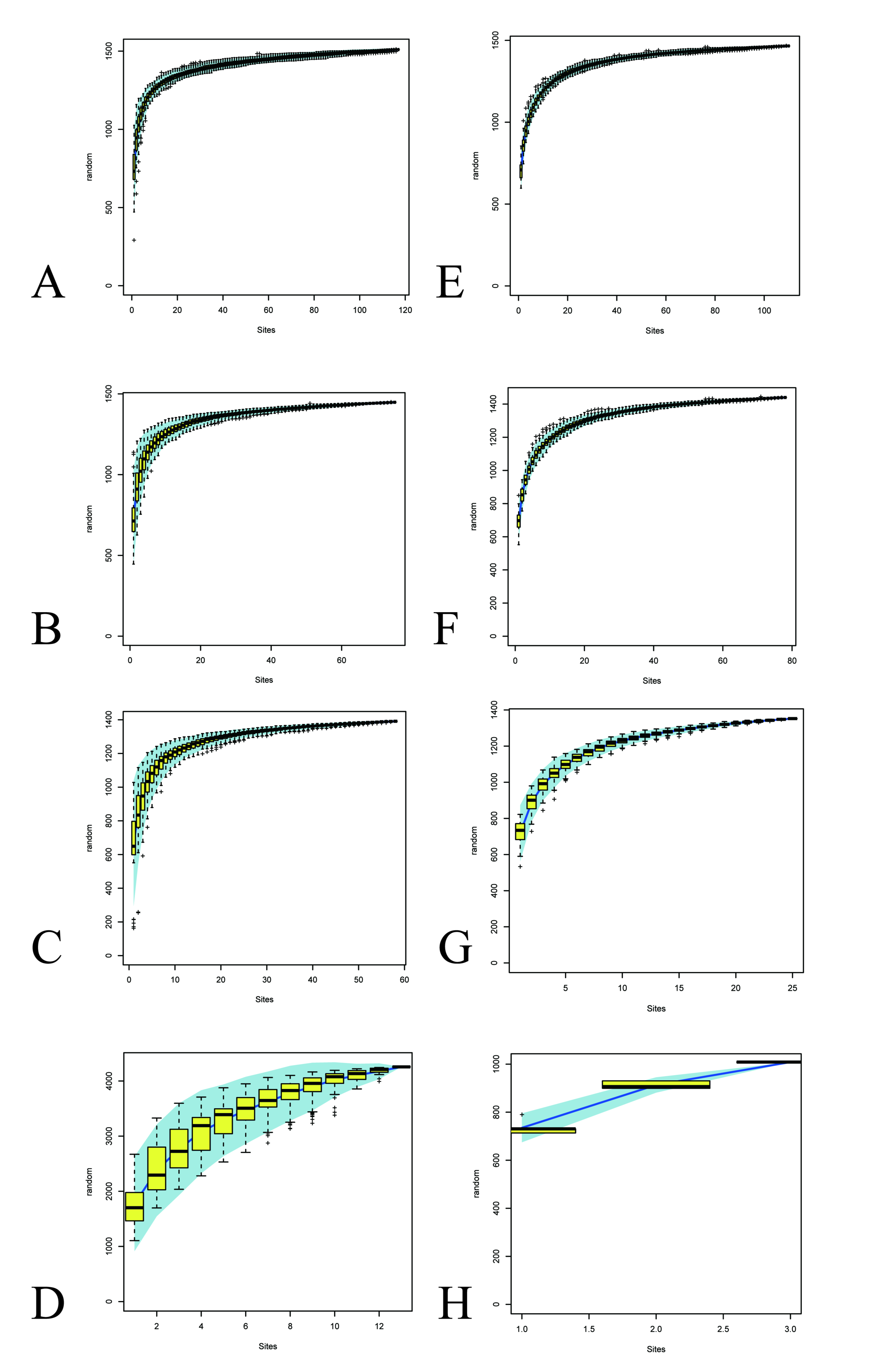

Supplement: Supplementary file 1 [file microorganisms-12-01351-s001.zip › Supplementary Figure S2.tif]

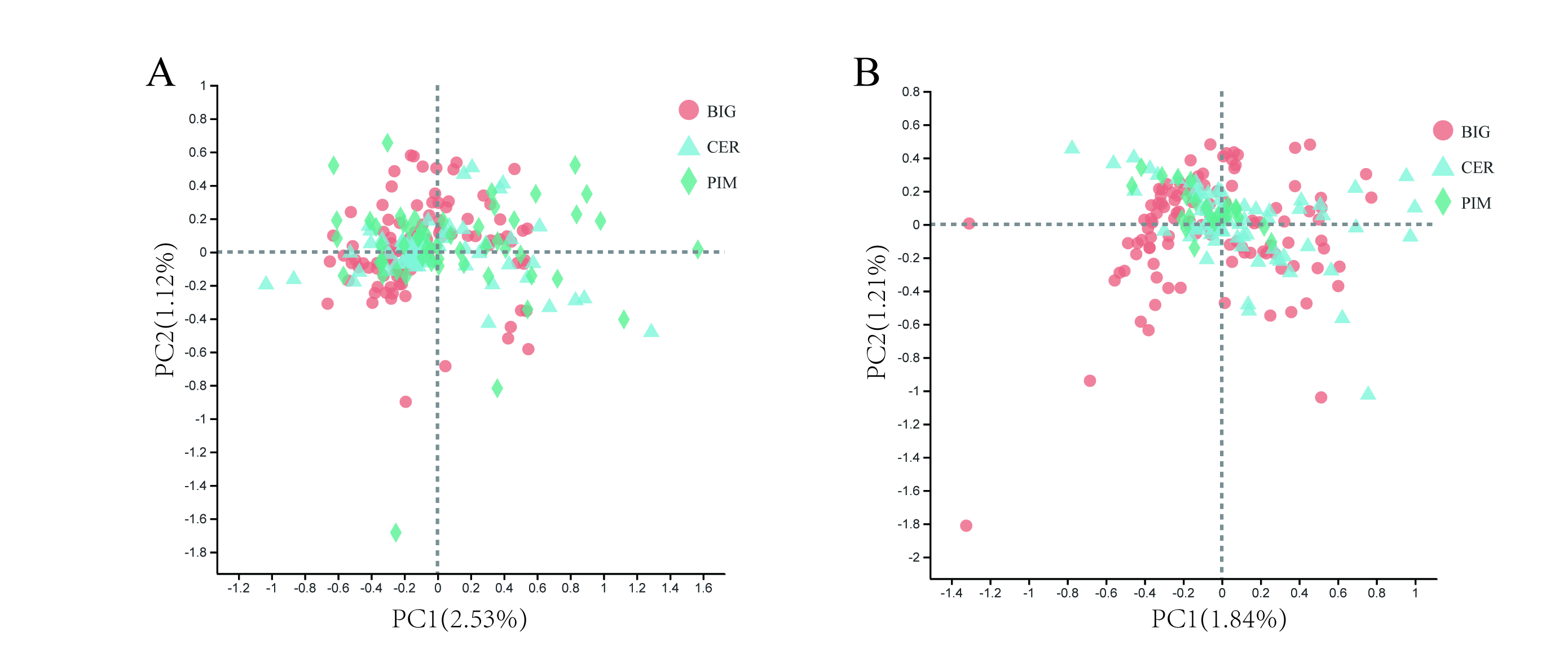

Supplement: Supplementary file 1 [file microorganisms-12-01351-s001.zip › Supplementary Figure S3.tif]

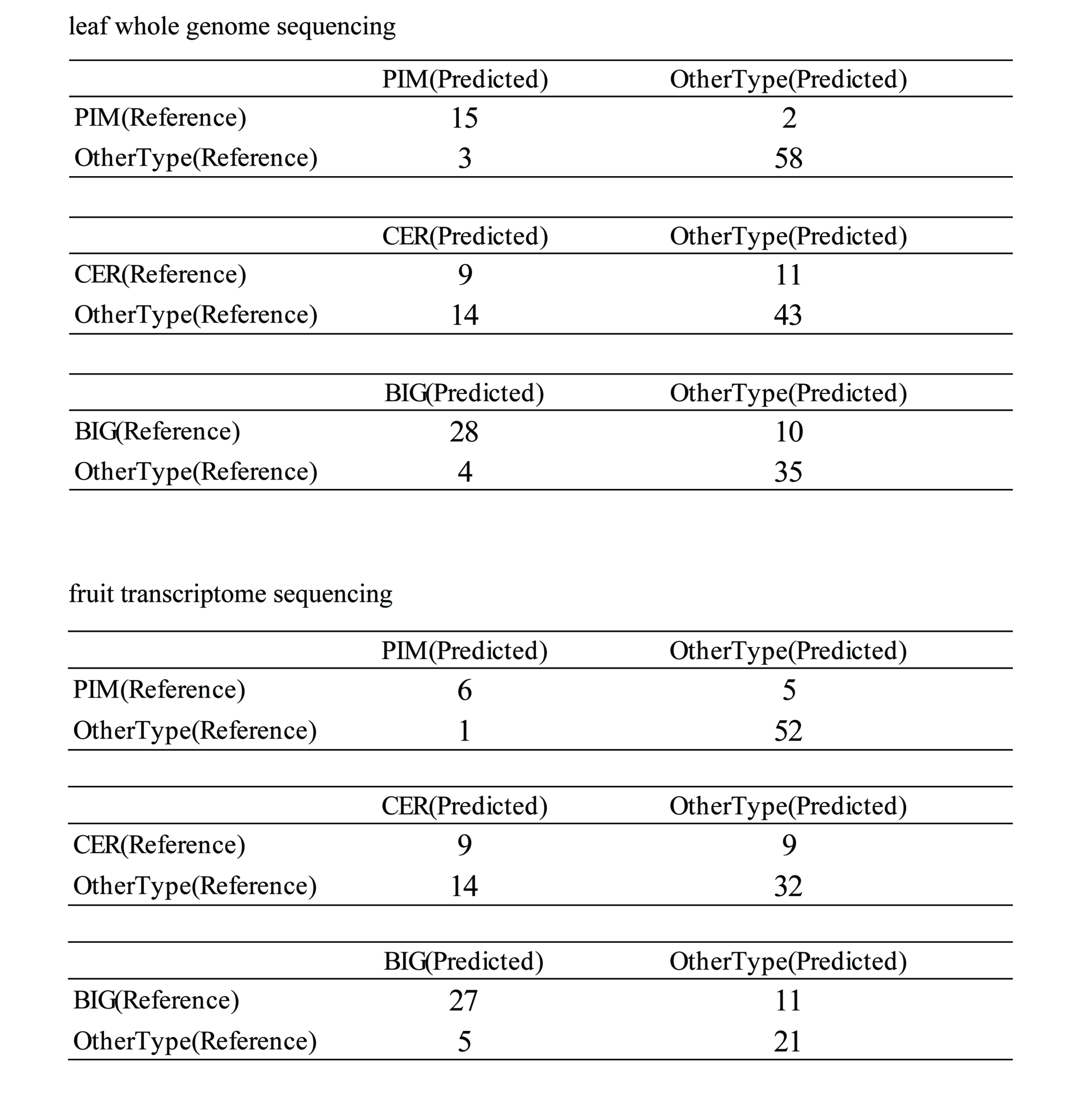

Supplement: Supplementary file 1 [file microorganisms-12-01351-s001.zip › Supplementary Figure S4.tif]

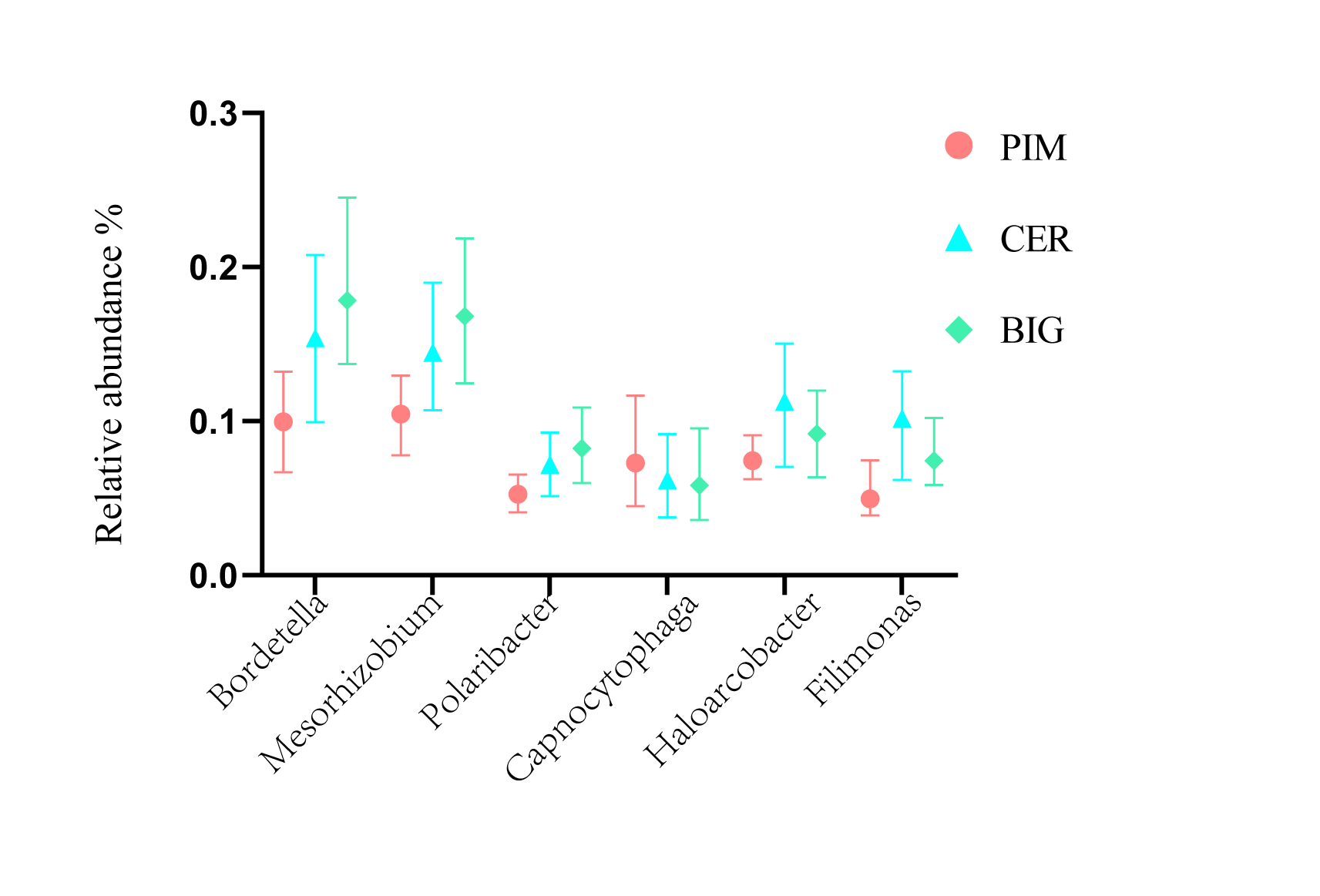

Supplement: Supplementary file 1 [file microorganisms-12-01351-s001.zip › Supplementary Figure S5.tif]
